# FEASIBILITY AND ACCEPTABILITY OF A VIRTUAL EXERCISE PROGRAM IN OLDER ADULTS WITH PREDIABETES

**DOI:** 10.1093/geroni/igae098.1029

**Published:** 2024-12-31

**Authors:** Kathryn Porter Starr, Nathan Boucher, Barbara Nicklas

**Affiliations:** Chair; Co-Chair; Discussant

## Abstract

Older adults undergo a decline in muscle mass by 30% between ages 50–70, resulting in increased risk of injury and even death, all of which can be prevented and managed with resistance training. Sarcopenia, aging-induced muscle loss, underscores resistance training’s critical role in muscle mass and function preservation. Despite the well-established benefits of resistance training, most older adults don’t meet this exercise threshold: only 19.3% of adults aged 65-74, and < 15% of older adults aged ≥75 report meeting the CDC guideline of performing moderate/high intensity muscle strengthening involving all major muscle groups on ≥2 days per week. Through a NIA-funded SBIR grant (R44AG076087) Vivo, a commercially based, virtual, live, and interactive exercise program, partnered with Duke University to examine the feasibility and acceptability of Vivo in older adults with prediabetes. In addition to building strength, the evidence-based program also incorporates dual task exercises to work on cognition, executive function, and processing speed. Sessions took place over zoom and were 45 minutes twice a week. This symposium provides an overview of Vivo, the feasibility study, perspective of a small business dedicated to improving the lives of older adults and a Vivo member. Both quantitative and qualitative program data on feasibility and acceptability for improving physical and psychosocial outcomes will be presented. Based on these initial findings, we believe Vivo shows great potential for reaching individuals with chronic illnesses who would benefit from adding strength training to their routines in a safe and accessible environment.

